# Prevention of cisplatin-induced nausea and vomiting by seabuckthorn (*Hippophae rhamnoides* L.) seed oil: Insights at the level of orexin-A in rats 

**DOI:** 10.22038/ijbms.2020.47599.10973

**Published:** 2021-02

**Authors:** Wen Yuan, Hongbo Wang, Yanling Gong

**Affiliations:** 1 Department of Pharmacy, College of Chemical Engineering, Qingdao University of Science and Technology, Qingdao, China; 2 Key Laboratory of Pharmaceutical Research for Metabolic Diseases, Qingdao University of Science and Technology, Qingdao, China; 3 Qingdao Jimo People’s hospital, Qingdao, China

**Keywords:** Cisplatin-induced vomiting, LHA, Orexin 1 receptor, Orexin-A, Seabuckthorn (Hippophae - rhamnoides L.) seed oil

## Abstract

**Objective(s):**

Nausea and vomiting are perennial problems in cancer patients undergoing chemotherapy. Orexin-A (OXA) has been shown to regulate feeding and gastric motility. Seabuckthorn (*Hippophae rhamnoides* L.) seed oil (SSO) has been proved to promote digestion and bowel movements. We investigated whether SSO alleviated cisplatin-induced vomiting and its possible mechanism involved in OXA.

**Materials and Methods:**

Rats were randomly divided into normal control group (NCG), cisplatin group (CG), SSO low-dose group (SLG), SSO middle-dose group (SMG) SSO high-dose group (SHG), and ondansetron group (OG). Rats were pretreated respectively with SSO (0.850, 1.675, and 3.350 g/kg·BW) and ondansetron (2 mg/kg·BW) in SLG, SMG, SHG, and OG for 6 days, and the same volume of saline in NCG and CG groups. On the 6th day, cisplatin (6 mg/kg, IP) was administered in all groups except NCG. The cumulative food and kaolin intake, gastric emptying, plasma OXA level, OX_1_R mRNA and protein expression in the hypothalamus and brainstem, and OXA expression in the lateral hypothalamic area (LHA) were observed, and the HPLC method was used to analyze the composition of SSO.

**Results:**

Kaolin intake in cisplatin-induced vomiting rats was significantly reduced (*P*<0.05) and gastric emptying delayed by cisplatin was improved (*P*<0.05-0.01) by pretreatment with SSO. Plasma OXA concentration, OX_1_R expression in the hypothalamus and brainstem increased significantly (*P*<0.05–0.01). Furthermore, OXA expression in LHA also increased significantly (*P*<0.05).

**Conclusion:**

SSO prevents cisplatin-induced vomiting in rats, which is possibly involved in increasing peripheral and central OXA and the expression of OX_1_R in the hypothalamus and brainstem.

## Introduction

Cisplatin-induced nausea and vomiting (CINV) is the main problem for cancer patients in the process of oncological treatment (1). Approximately half of the cancer patients experience nausea or vomiting, either because of chemotherapy or cancer itself. There are three kinds of vomiting induced by chemotherapy: acute vomiting, delayed vomiting, and early vomiting. Patients who received cisplatin, carboplatin, or cyclophosphamide chemotherapy are prone to delayed vomiting. Cisplatin chemotherapy damages the gastrointestinal tract and causes enterochromaffin cells in the gastrointestinal mucosa calcium-dependent efflux 5-hydroxytryptamine (HT)3. The released 5-HT3 combines with receptors on the vagus afferent neurons to activate the chemoreceptor trigger area (CTZ) and the vomiting center (VC). When CTZ is stimulated, it also activates VC by releasing various neurotransmitters in turn. Once activated, VC regulates transmission to the esophagus, leading to vomiting (2). There are several neurotransmitters involved in the pathogenesis of emesis such as serotonin, dopamine, histamine, and substance P. Recent studies have shown that orexin-A (OXA) is associated with regulation of gastrointestinal function (3). So OXA may also be related to vomiting in cisplatin-treated mice.

Orexins/hypocretins are regarded as a novel neuropeptide which is mainly located in the lateral neurons of the hypothalamus (4). Orexin nerve fibers are widely projected into the central nervous system including the paraventricular nucleus (PVN), the central medial nucleus, brain stem, and spinal cord. OXA and orexin-B (OXB) constitute the orexin neuropeptide family and both are produced in the hypothalamus by an enzymatic cascade of propeptide across the brain (5). OXA, made up of 33 amino acids, activates the orexin-1 receptor (OX_1_R) and orexin-2 receptor (OX_2_R) in a similar way (6). OXA stimulates the vagus motor neurons of the gastric projection (7). OXA administration in the brain induced proximal gastric relaxation, meanwhile it promoted distal gastric contraction in anesthetized rats. Both OXA induced relaxation and contraction were weakened by vagotomy (8). A recent study has shown that OXA is closely associated with gastric motility through the lateral hypothalamic area (LHA) (9). It is speculated that an increase of OXA or activation of OX_1_R might be beneficial to promote gastric motility.

Seabuckthorn (*Hippophae rhamnoides *L.) seed oil (SSO), extracted from seeds of plants in a group of species of the genus *Hippophae*, is widely used in dietary supplements, nutraceuticals, cosmetics, and skincare products (10). SSO contains a large amount of unsaturated fatty acids, tocopherol, carotenoids, and flavonoids, which have significant anti-bacterial, anti-oxidation, anti-atherosclerosis, and cardioprotective activities (11). In addition, SSO can also promote digestion and bowel movements. A study shows that seabuckthorn flesh and seed oil can reduce apoptosis and inflammation, thus protecting the intestinal tract of mice from high-dose radiation (12). Therefore, we hypothesized whether SSO can alleviate vomiting induced by cisplatin. In addition, vitamin E is a natural antioxidant in SSO. The higher the content, the more stable the oil. The high-performance liquid chromatography (HPLC) analysis of vitamin E can be used to detect the stability and authenticity of SSO. 

In this study, we are dedicated to exploring the effect of SSO on cisplatin-induced vomiting in rats. HPLC was used to analyze the components of SSO, and vitamin E was taken as the main index for the quality control of SSO. The cumulative food and kaolin intake, gastric emptying, plasma OXA level, expressions of OX_1_R mRNA and protein in the hypothalamus and brainstem, OXA expression in the LHA were detected in cisplatin-induced vomiting rats after peripheral administration of SSO to reveal the potential mechanism. 

## Materials and Methods

***Animals and reagents***

***Animals***

Healthy male Wistar rats weighing about 250 g were purchased from Qingdao Daren fortune animal science and technology co. LTD. Rats were raised in individual cages under controlled 22±2 °C environmental conditions (12 hr dark/light cycles, relative humidity 40–60%, food and water *ad libitum*). All rats were adaptively fed for 7 days before the experiment. All animal experiments conducted by the laboratory are in compliance with the regulations of Qingdao University of Science and Technology and approved by the animal ethics committee of Qingdao University of Science and Technology. 

Reagent 

SSO (>98% purity) was purchased from Qinghai Qinghua Bozhong biotechnology co., Ltd. (Qinghai, China). Ondansetron was purchased from Qilu pharmaceutical co. Ltd. (Shandong, China). Dl-α-tocopherol acetate was obtained from Beijing Solarbio Science & Technology Co., Ltd. Methanol (gradient grade for HPLC) was obtained from Sigma-Aldrich (St. Louis, MO, USA). 

*Preparation of kaolin feed*

Kaolin feed was prepared according to the method of the literature (13) with minor modifications. 50 g kaolin and 2% Arab powder were mixed, a moderate amount of distilled water was added to soften the material. Then it was rubbed into of columnar like the index finger or thumb by hands, placed in 45 °C oven until dry. In order to ensure the credibility of the control experiment, the normal feed should be prepared into the same shape in the same way. 

Semisolid paste preparation 

8 g milk powder was dissolved in 125 ml distilled water and the following ingredients were added: 5 g hydroxymethyl cellulose, 4 g glucose, and 4 g starch, 1 g activated carbon, and mixed to make 150 ml semisolid paste (150 g) (14). The semisolid paste was stockpiled in the refrigerator for 12 hr and put at room temperature for 2 hours before use.

***Group and treatments***

Rats (N=108) were randomly divided into 6 groups: the normal control group (NCG), cisplatin group (CG), ondansetron group (OG), SSO low dose group (SLG), SSO medium-dose group (SMG), and SSO high dose group (SHG). The rats in SLG, SMG, SHG and OG groups were intragastrically administrated with 0.4 ml of SSO with different concentrations (0.850 g/kg·BW, 1.675 g/kg·BW, 3.350 g/kg·BW) and ondansetron (2 mg/kg·BW) for 6 days, respectively. The rats in the NCG group and CG group were both given an equal volume of normal saline. On the 6th day after administration, the rats in the NCG group were intraperitoneally injected with normal saline while 6 mg/kg cisplatin was intraperitoneally injected to those in the remaining five groups (15). All the rats were given a quantitative and adequate amount of water, normal feed, and kaolin. After 72 hr, the total food intake and kaolin consumption of rats were recorded, and the quantity of kaolin consumption was used to evaluate the pica of rats.

***Gastric emptying test***

Eight rats in each group were fasted for 18 hr and given intragastrically 0.4 ml semisolid paste 40 min after the last administration. Then, the rats were anesthetized with intraperitoneal injection of phenobarbital (8 mg/kg), and the abdominal cavity was opened, the cardia and pylorus were ligated. The stomach was removed and weighed after being desiccated with filter paper. Then it was cut off along the greater curvature. The stomach contents were washed clean and wiped to dry to record the net weight. The gastric emptying rate was calculated using the following formula (14): 

Gastric emptying rate (%) = [1−(stomach full weight -net weight )]´100% 

***Collection of plasma, hypothalamus, and brainstem***

Eight rats in each group were used to collect blood and fresh brain tissues. After anesthesia with intraperitoneal injection of pentobarbital (8 mg/kg), blood was taken from the heart to separate plasma and stored at -80 °C for further analysis. Simultaneously, the brain was quickly isolated on 4 °C ice, hypothalamus and brainstem were separated carefully and flash-frozen at -80 °C for subsequent mRNA and protein detection. Brain regions were separated according to the Atlas of Paxinos and Watson (16). The central point between the gray nodules and the optic chiasm was taken as the center to determine the hypothalamic tissue (the anterior border of the optic chiasm is the anterior border, the posterior border of the papillary body is the posterior border, and the lateral temporal sulcus is on both sides, the overall width is about 4 mm, the depth is about 2 mm, and the length is about 4 mm). The brainstem is taken according to the following boundary: the caudal end is the foramen magnum, the ventral end of the cephalic end is the lower margin of the diencephalon, and the dorsal end of the cephalic end is the upper margin of the superior colliculus, as shown in Figure S1. 

***Perfusion fix of brain***

Two rats of each group were anesthetized with pentobarbital (8 mg/kg). Perfusion was performed with normal saline followed by 4% paraformaldehyde. The brains were stripped and fixed in 4% paraformaldehyde for 6~8 hr. Subsequently, they were removed to a 30% sucrose solution until the brains sank. Each brain was sliced continuously into 15 μm thick frontal sections on a freezing microtome (Kryostat 1720, Leica, Germany). 

Detection of orexin-A concentration in the plasma 

The plasma OXA level was measured using ELISA kits (Phoenix Pharmaceuticals, CA, USA). 

Detection of OX_1_***R mRNA in the hypothalamus and brainstem by quantitative real-time polymerase chain reaction ***

The hypothalamus and brainstem samples were treated with Trizol Plus RNA Purification Kit (Invitrogen, CA, USA). Total RNA was extracted according to the instructions provided by the manufacturer. 1 μg of total RNA was reverse transcribed using the First Strand cDNA Synthesis Kit (Pharmacia Biotech, Piscataway, NJ, USA). The target gene primer sequence of OX_1_R was shown in Table 1. The reaction was accomplished under the following conditions: denaturation for 10 min at 95 °C, 95 °C last 15 sec, circulating 40 times, and 1 min of annealing at 60 °C. 

SYBR Green master mixsequence detector (Applied Biosystems, UK) was used to conduct real-time PCR. Gene Amp 5700 SDS software (Applied Biosystems, Warrington, UK) was used to analyze the data. Relative quantification was conducted by counting the threshold cycle difference between OX_1_R and β-Actin (ΔCt=Ct_OX1R_−Ct_β_-_Actin_).ΔΔCt=ΔCt_Experiment_−ΔCt_Control_.

The threshold cycle of OX_1_R mRNA expression between the experiment and control groups is equal to 2^−ΔΔCt^. 

***Detection of OX***_1_ ***R protein in the hypothalamus and brainstem by Western blot***

The hypothalamus and brainstem samples were respectively placed in 1 ml frozen cell lysate for 30 min to dissolve the cells uniformly. Then, it was centrifuge washed 15 min at 14000 r/min at 4 °C, and protein concentration was determined by a second quinoline formic acid test. 50 μg of proteins were placed in 10% sodium dodecyl sulfate-polyacrylamide gel (SDS-PAGE). The proteins were moved to polyvinylidene fluoride (PVDF) membranes (Millipore, Billerica, MA, USA) for 2 hr after electrophoresis, and sealed with 5% skim milk for 1 hr in the wash buffer (TBST, 10 mM Tris hydrochloric acid buffer, pH=7.5, 0.1% tween 20, 150 mmol/l NaCl). Then blots were incubated overnight in primary OX_1_R antibodies (rabbit polyclonal, 1:1000 dilution, Abcam, USA) at 4 °C. All transfer films were washed 5 times in TBST for 10 min each time. Then it was incubated with horseradish peroxidase-conjugated anti-rabbit IgG for 2~3 hr at room temperature and washed 5 times in TBST for 10 min each time. Proteins were tested with enhanced chemiluminescence (ECL) reagent kit (Bioss, Beijing, China) and radiographic films (Bioss, Beijing, China). To monitor the loading of gel lanes, the blots were stripped and β-actin antibody was used to re-probe. 

***Immunohistochemistry for orexin-A in LHA***

The sections were treated with citrate buffer and 0.3% H_2_O_2_ and then sealed with 5% goat serum for 1 hr at room temperature. At the same time, the sections were hatched with a rabbit polyclonal antibody for OXA (Abcam, USA, diluted at 1:200) in a diluent solution containing 4% goat serum and 0.2% Triton X-100 in PBS at 4 °C overnight. In subsequence, they were washed 3 times in PBS. Next, the sections were incubated with Cy3 labeled goat anti-rabbit antibody (Bioss, Beijing, China, diluted at 1:200) for 1 hr at room temperature on the following day and then washed 3 times in PBS. Photos were taken under a BX50 microscope and a DP50 digital camera (Olympus, Tokyo, Japan). 

***High-performance liquid chromatography (HPLC) for SSO***

Two g SSO was put into a Pyrex tube. The sample was saponified by adding KOH (60%, w/v), anhydrous ethanol, and ethanol pyrogallol (6%, w/v) and then adding normal saline. Tocopherol was extracted from the mixture with hexane/ethyl acetate (9/1,v/v). The upper transparent clarifying liquid was placed in the test tube and evaporated to dry in the oven. The drying residue was dissolved with hexane/isopropanol (99/1, v/v), and placed in the dark at 4 °C until analyzed. 

The chromatographic analysis was performed on a Waters e2695 HPLC system with a manual injector of 10 μl sample loop and a UV-Vis detector. The data were integrated using Empower 3 software. Dl-α-tocopherol acetate was chosen as the standard. All separations were carried out on an MP C18 column (100 mm × 4.6 mm, with 3.5 μm particle size) from Agilent. The column temperature was 30 °C. The mobile phase consists of methanol and water (98:2) and the flow rate was controlled at 1 ml/min for isometric elution. All measurements were performed at a wavelength of 280 nm. 

***Statistical analysis***

Results are presented as the mean±standard deviation and analyzed by one-way analysis of variance (ANOVA) and LSD test using SPSS software, Version 17.0 (SPSS Inc., Chicago, IL, USA). A *P* value of 0.05 or less was considered statistically significant. 

## Results

***Effects of SSO on cumulative food intake and kaolin intake in cisplatin-treated rats***

**Figure 1:**
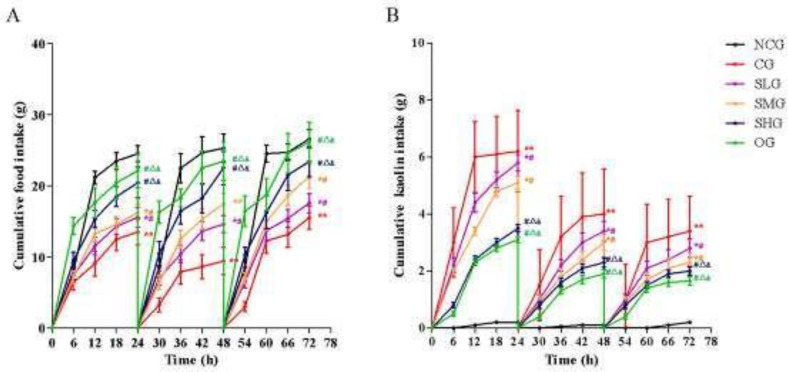
The effects of seabuckthorn seed oil (SSO) on cisplatin-induced anorexia (A) and pica (B) in rats. SSO increased food intake and decreased kaolin intake in a dose-dependent manner (n=18). *P<0.05, **P<0.01 versus normal control group (NCG); #*P*<0.05 versus cisplatin group (CG); ^△^ *P*<0.05 versus SSO low dose group (SLG); & *P*<0.05 versus SSO medium dose group (SMG)

The cumulative food intake and kaolin intake of rats were recorded 6-hourly for 72 hr after cisplatin administration. The cumulative food intake in the CG group decreased while kaolin intake increased 6 hr after cisplatin administration and lasted for 72 hr, indicating development of pica induced by cisplatin (versus the NCG group, *P*<0.01, Figures 1A and 1B). Pica was attenuated to varying degrees after administration of different doses of SSO (versus the CG group, *P*<0.05, Figures 1A and 1B). Furthermore, there was no significant difference between the SHG group and the OG group (*P*>0.05, Figures 1A and 1B). 

***Effects of SSO on gastric emptying in cisplatin-treated rats***

**Figure 2:**
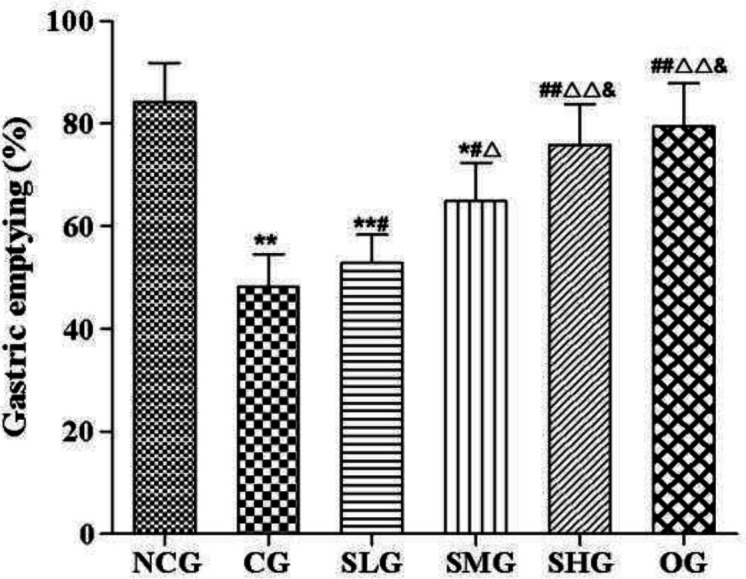
The effect of seabuckthorn seed oil (SSO) on gastric emptying in cisplatin treated rats (n=8). * *P*<0.05, ** *P*<0.01 versus normal control group (NCG), # *P*<0.05, ## *P*<0.01 versus cisplatin group (CG); ^△^ *P*<0.05, ^△△^ P<0.01 versus SSO low dose group (SLG); & *P*<0.05 versus SSO medium dose group (SMG)

In the present study, cisplatin intraperitoneally injected resulted in a delay of gastric emptying (*P*<0.01, Figure 2). After intragastric administration of SSO, gastric emptying increased significantly in varying degrees when compared with the CG group (*P*<0.05-0.01, Figure 2). The SHG group showed no statistical difference in gastric emptying rate with the OG group (*P*>0.05, Figure 2). 

***Effects of SSO on plasma OXA in cisplatin-treated rats***

**Figure 3:**
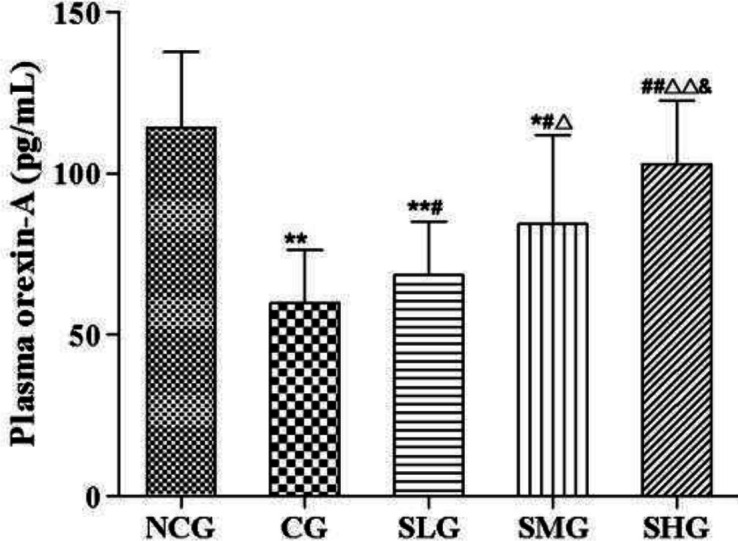
The effect of seabuckthorn seed oil (SSO) on plasma OXA in cisplatin treated rats (n=8). * *P*<0.05, ** *P*<0.01 versus normal control group (NCG); # *P*<0.05, ## *P*<0.01 versus cisplatin group (CG); ^△^ *P*<0.05, ^△△^ *P*<0.01 versus SSO low dose group (SLG); & *P*<0.05 versus SSO medium dose group (SMG)

The changes of OXA level in plasma were detected. Intraperitoneal injection of cisplatin significantly decreased the levels of OXA in plasma compared with those in the NCG group (*P*<0.01, Figure 3). However, the decreases were significantly reversed by intragastrical treatment with different doses of SSO (versus the CG group, *P*<0.05–0.01, Figure 3). 

***Effects of SSO on mRNA expression of OX***_1_ ***R in the hypothalamus and brainstem***

**Figure 4:**
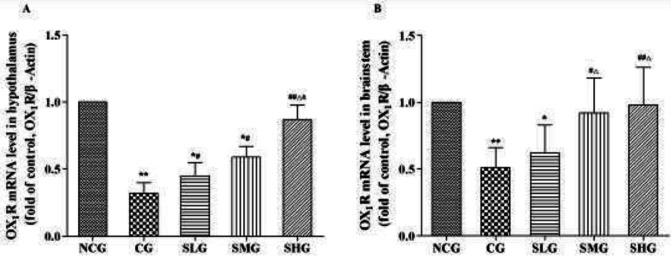
The effect of seabuckthorn seed oil (SSO) on mRNA expressions for OX1R in the hypothalamus (A) and brainstem (B) of cisplatin-treated rats (n=8). * *P*<0.05, ** *P*<0.01 versus normal control group (NCG); # *P*<0.05, ## *P*<0.01 versus cisplatin group (CG); ^△^ *P*<0.05 versus SSO low dose group (SLG); & *P*<0.05 versus SSO medium dose group (SMG)

According to the RT-PCR results in Figures 4A and 4B, the expression of OX_1_R mRNA in the hypothalamus and brainstem in the CG group was obviously lower than in the NCG group (*P*<0.01). Surprisingly, SSO increased the expression of OX_1_R mRNA in the hypothalamus and brainstem, especially in the SHG group (*P*<0.05–0.01, Figures 4A and 4B). 

***Effects of SSO on protein expression of OX***_1_ ***R in the hypothalamus and brainstem***

**Figure 5:**
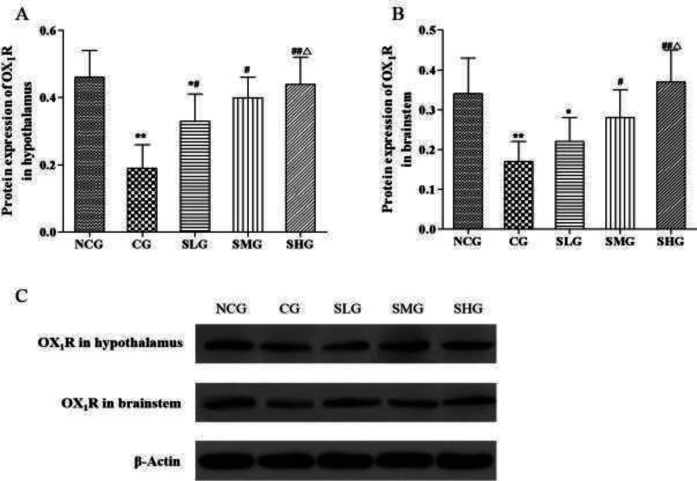
The effect of seabuckthorn seed oil (SSO) on protein expression of OX1R in the hypothalamus (A) and brainstem (B) of cisplatin treated rats (n=8). *P<0.05, ** *P*<0.01 versus normal control group (NCG); #*P*<0.05, ## *P*<0.01 versus cisplatin group (CG); ^△^*P*<0.05 versus SSO low dose group (SLG); & *P*<0.05 versus SSO medium dose group (SMG)

The expression of OX_1_R protein was also detected in our present study. As revealed in the results, cisplatin decreased the expression of OX_1_R protein both in the hypothalamus and brainstem (*P*<0.05–0.01, Figures 5A and 5B). After SSO administration, the expression of OX_1_R protein increased significantly both in the hypothalamus and brainstem (*P*<0.05–0.01, Figures 5A and 5B) to a different degree (*P*<0.05–0.01, Figures 5A and 5B). 

***Effects of SSO on OXA expression in the LHA***

**Figure 6:**
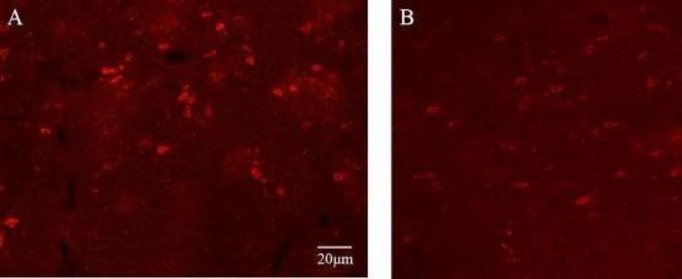
The effect of seabuckthorn seed oil (SSO) on expression of OXA neurons in the LHA of cisplatin-treated rats A: normal control group (NCG); B: cisplatin group (CG); C: SSO high dose group (SHG). Scale bar indicates 20 μm

The expression of OXA in the LHA was detected by immunohistochemical analysis. There were OXA responsive neurons in the LHA of rats in the NCG group (Figure 6A). However, the expression of OXA neurons in the LHA was decreased by cisplatin (Figure 6B), which was reversed by a high dose of SSO (Figure 6C). 

***Results of HPLC for seabuckthorn seed oil***

**Figure 7:**
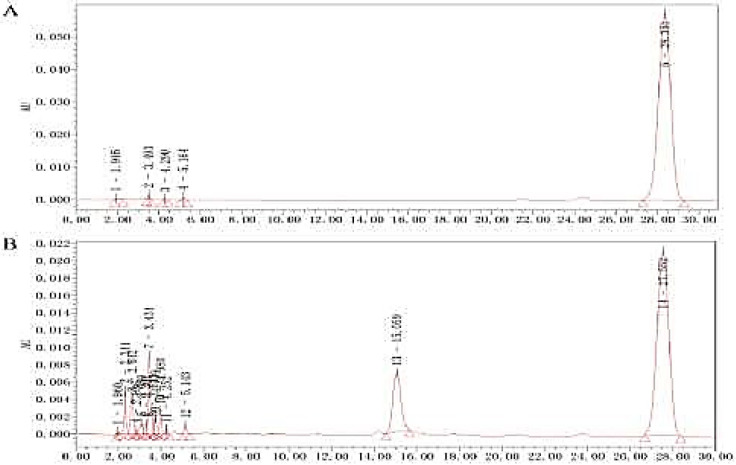
HPLC analysis for Seabuckthorn seed oil. A. dl-α-tocopherol acetate; B. seabuckthorn seed oil

The results of HPLC analysis of dl-α-tocopherol acetate indicated that its peak was eluted at about 28 min (Figure 7A). As can be seen from Figure 7B, the above SSO contains 14 components and is rich in vitamin E. Therefore, SSO is a good source of vitamin E and high-quality health care vegetable oil. 

**Table 1:** The primers used in real-time RT–PCR

**Primers **	**Sequence **
OX_1_R	Forward: 5’-GGACCACTGCACCGAAGA-3′ Reverse: 5’-GGTTACCGTTGGCCTGAA-3′
β-Actin	Forward: 5’-CACCCTGTGCTGCTCACCGAGGCC-3′ Reverse: 5’-CCACACAGATGACTTGCGCTCAGG-3′

## Discussion

This study clarified the effect of SSO on the vomiting behavior of rats induced by cisplatin and its possible mechanism. The results revealed that SSO alleviated 

CINV in rats and improved gastric emptying. Furthermore, SSO elevated plasma OXA concentration and promoted OX_1_R expression in the hypothalamus and brainstem as well as OXA expression in LHA, which might be related to its anti-vomiting effect.

Seabuckthorn, one of the plants of the *Elaeagnaceae* family, is a deciduous, thorny, nitrogen-fixing plant native to Eurasia and has been spread in many countries including Russia and China. Every part of seabuckthorn, especially the pulp and seeds, have been used as ingredients for nutritive foods and therapeutic properties (17). In China and Russia, Seabuckthorn pulp and seeds have been used for centuries as herbal remedies for burns and wound healing, cough suppressant, or digestive aid. SSO contains a high proportion of essential unsaturated fatty acids such as oleic acid, palmitic acid, and linoleic acid. Besides, it also contains tocopherols, phytosterols proanthocyanidin (GP), and carotenoids, ponderable bioactive substances that can be obtained from products considered waste (18). High levels of β-carotene, β-sitosterol, and α-tocopherol have been shown to have protective activity on the stomach (19). Proanthocyanidins are antioxidant flavonoids that not only have neuroprotective effects in humans and animals but also reduce pica behavior (modeled on non-edible substances) (20,21). Meanwhile, it has been shown that seabuckthorn seed and pulp oil are effective in the prevention and treatment of gastric disease in rats (22). And the seed oil can treat diseases such as gastric ulcers induced by dexamethasone (23). To our knowledge, it is the first time to report the effective function of SSO on the CINV. Our results revealed that SSO inhibited the kaolin intake, increased food intake, and promoted gastric emptying in the cisplatin-treated rats. 

Nausea and vomiting are a complex body defense mechanism. Nevertheless, when these phenomenon are repeated frequently, they can significantly affect the quality of life and may be detrimental to health when repeated frequently (24). Chemotherapy is a common treatment for many cancer patients, but it is often accompanied by severe nausea and vomiting. In our present study, increase of kaolin intake indicated nausea and vomiting induced by cisplatin. Although cisplatin is effective to treat cancer, it causes the formation of free radicals in the intestine that release 5-HT (25). This release of 5-HT relaxes the stomach by acting on the 5-HT receptor, resulting in a delay in gastric emptying, which reflects the accumulation of food substances in the gastric and leads to vomiting (26). Although advances have been achieved in resistance to vomit treatment, chemotherapy-induced emesis remains a significant burden for chemotherapy patients (27). It has been reported that inhibition of ileum contraction in guinea pigs by acting on the 5-HT_3_ receptor may be related to its antiemetic activity (28). Inhibiting the increase of dopamine (DA) in the peripheral and central systems by inhibiting dopamine D2 receptor (D2R), tyrosine hydroxylase (TH), and promoting dopamine transporter (DAT) is beneficial to CINV (29). OXA is a recently revealed hypothalamic neuropeptide of 33-amino acids that affects gastrointestinal motility, promotes eating, and increases body weight (30). Whether it is involved in CINV is under exploration. 

OXA is an important peptide related to food intake. OXA neurons are mainly expressed in the regions of the lateral hypothalamus, lateral dorsal side, and around the fornix. OX_1_R, the main receptor of OXA, is widely expressed in the central nervous system, especially the hypothalamus and brainstem. It is reported that intracisternal administration of OXA in rats relaxed the proximal stomach and facilitated the motility of the antral portion via the vagus nerves (31). In our present study, a decrease of plasma OXA, OX_1_R expression in the hypothalamus and brainstem, OXA expression in the LHA was observed in the cisplatin-treated rats. The above finding indicated destruction of the OXA system by cisplatin, which might be involved in the pathogenesis of the CINV. Kaolin intake is commonly used to assess cisplatin-induced pica and vomiting in rats. Antiemetic effects of newly developed medicines in preclinical studies have illustrated that OXA can also influence the increased kaolin intake induced by cisplatin (32). OXA involvement in the arcuate nucleus (ARC)-paraventricular nucleus (PVN) circuitry in the brain promoted gastric motility to mitigate the emesis induced by cisplatin (33). 

Based on the above findings, we have explored the underlying mechanism of SSO in the treatment of CINV. Amazingly, a potential role involved in OXA has been revealed in our present study. The increase of plasma OXA level after SSO pretreatment in cisplatin-induced vomiting rats may be related to the acceleration of gastric emptying. Furthermore, the expression of OX_1_R mRNA and protein in the hypothalamus and brainstem and OXA in the LHA increased after treatment with SSO, indicating a central effect of SSO. The reason may be that proanthocyanidins and total flavonoids in SSO can regulate the expression of OXA in LHA through the blood-brain barrier. However, the specific mechanism needs further study. 

## Conclusion

SSO alleviates vomiting induced by cisplatin and promotes gastric emptying, increases plasma OXA, elevated OX_1_R expression in the hypothalamus and brainstem, and OXA expression in the LHA. Our present study sheds light on a possible peripheral and central role involved in OXA of SSO effect on the CINV. These findings can offer a viable therapeutic mechanism and strategy for the side effects concerned with a specific tumor or cancer chemotherapy drug. 
